# The term “physical distancing” is recommended rather than “social distancing” during the COVID-19 pandemic for reducing feelings of rejection among people with mental health problems

**DOI:** 10.1192/j.eurpsy.2020.60

**Published:** 2020-06-01

**Authors:** Danuta Wasserman, Rutger van der Gaag, Jan Wise

**Affiliations:** 1 Psychiatry and Suicidology; World Health Organization’s (WHO) Lead Collaborating Centre for Research, Developments, and Training in Suicide Prevention at Karolinska Institutet, Stockholm, Sweden; 2 European Psychiatric Association (EPA) Ethics Committee, Brussels, Belgium; 3 Psychosomatics and Psychotherapy, Riga Stradina University, Riga, Latvia; 4 Adult General Psychiatry CNWL NHS Foundation Trust, London, UK

## Abstract

As COVID-19 has plagued our world, the term “social distancing” has been widely used with the aim to encourage the general population to physically distance themselves from others in order to reduce the spread of the virus. However, this term can have unintended but detrimental effects, as it evokes negative feelings of being ignored, unwelcome, left alone with one's own fears, and even excluded from society. These feelings may be stronger in people with mental illnesses and in socio-economically disadvantaged groups, such as stigmatized minorities, migrants, and homeless persons [1], many of them also having high risk for suicidal behaviors [2]. Mental health disorders are pervasive worldwide; the global burden accounting for approximately 21.2–32.4% of years lived with disability—more than any other group of illnesses [3]. So, the vulnerable group of people with mental health disorders represents a considerable share of the total global population.

As COVID-19 has plagued our world, the term “social distancing” has been widely used with the aim to encourage the general population to physically distance themselves from others in order to reduce the spread of the virus. However, this term can have unintended but detrimental effects, as it evokes negative feelings of being ignored, unwelcome, left alone with one's own fears, and even excluded from society. These feelings may be stronger in people with mental illnesses and in socio-economically disadvantaged groups, such as stigmatized minorities, migrants, and homeless persons [1], many of them also having high risk for suicidal behaviors [2]. Mental health disorders are pervasive worldwide; the global burden accounting for approximately 21.2–32.4% of years lived with disability—more than any other group of illnesses [3]. So, the vulnerable group of people with mental health disorders represents a considerable share of the total global population.

Suicidal ideation is common among a broad group of patients with mental problems, including affective, psychotic and anxiety disorders. People with suicidal ideation have a pessimistic and dark outlook on life, and may be very sensitive to any signs of rejection. The suicidal process may be accelerated due to an interplay between the receivers’ desperate and chaotic feelings and the communication that “social distancing is necessary” circulating throughout every level of society. The inner psychological entrapment of persons close to suicide [4] can be magnified not only by physical entrapment in the lockdown situations, but also by the terminology used of social distancing. In individuals at risk of psychotic breakdowns, the appearance of face masks and goggles is likely to induce distrust easily shifting into paranoia. Negative emotions, evoked by social distancing connotations, are unfortunately all too real for the beholder and have a crucial bearing on vulnerable persons, especially those who are prone to suicidal actions and ambivalent to living or dying [2]. During these unprecedented times, filled with anxiety about COVID-19 consequences, emotional closeness, connection, and inclusivity are increasingly important [5].

In times of crisis, there are centrifugal movements among the general public. On one side, we see solidarity and more care for each other; on the other side, sadly prejudice reigns. The term social distancing may enhance latent racism or xenophobia, especially against migrant groups and refugees [6]. It may also increase the isolation of children and women, who are prone to domestic violence [7]. In all these groups, the term social distancing, as well as the fact that they are closely locked-down with their potential aggressors with reduced opportunity for being helped by others, is strongly pathogenic. Moreover, in many vulnerable groups and in frail elderly, the lockdown may induce or enhance loneliness and feelings of being a burden to their dear ones and society in general. This can lead to feelings of depression and eventually suicidal thoughts and acts [8].

The notion of “social distancing” affords neither comfort nor help to vulnerable people. They are often reliant on social connection and relations, so a sudden break in those, perceived or real, has devastating consequences. Therefore, we advocate to use the term “physical distancing”—as a physical distance of 2 m will prevent and diminish the spread of COVID-19—rather than the term “social distancing” [1].

While the large majority of the general population are unlikely to react negatively to the term of social distancing, it is important that the majority is informed and aware of how vulnerable groups in society perceive and react to this term. The literacy of the general population about mental health is unfortunately still very low, as well as the knowledge about how it is possible for lay people to support persons with mental health problems [9].

As psychiatrists, along with other mental health professionals, we must embrace, alongside our daily tasks of early detection, treatment, and rehabilitation of persons with mental disorders, the job of increasing public mental health awareness and collaborating with public health institutions by giving information, advice, and guidelines [10]. We should draw to their attention the benefits of phone contact, the options for basic psychological support, and the availability of stepped care whether online support, an app or remote support, and/or treatment from a community service [11].

Now, as the world begins to reduce the restrictions in place and return to some sense of normality during the ongoing COVID-19 pandemic, daily social interactions will increase. However, also during this time, everyone should be careful about maintaining a physical distance to avoid a second wave of COVID-19 infections—yet at the same time remain social, supportive, and empathetic toward one another. Let us be inclusive, in both our words and our actions, so that no one feels excluded or isolated from their wider community.

After one of the authors who is also the Chair of the EPA Ethics Committee (D.W.) alerted the WHO Mental Health and Substance Abuse division that the term “social distancing” was adversely influencing vulnerable groups, the WHO’s COVID-19 response team on March 23 addressedthe media stating that the term “physical distancing” will replace “social distancing” for the sake of mentally ill persons.

Policy makers, media, governments, and the general public should be encouraged to use the more neutral term of “physical distancing” rather than “social distancing” during the COVID-19 pandemic on the basis of the negative connotations of this term.
